# Formation of Silver Nanoparticles Using Fluorescence Properties of Chitosan Oligomers

**DOI:** 10.3390/md16010011

**Published:** 2018-01-03

**Authors:** Ja Young Cheon, Hun Min Lee, Won Ho Park

**Affiliations:** Department of Advance Organic Materials and Textile System, Chungnam National University, Daejeon 34134, Korea; alranim@nate.com (J.Y.C.); hun1062@naver.com (H.M.L.)

**Keywords:** chitosan oligomer, silver chloride (AgCl), nanoparticles, fluorescence, quenching

## Abstract

In this study, silver chloride nanoparticles (AgCl NPs) were prepared using chitosan oligomer (CHI) and chitosan oligomer derivatives (CHI-FITC). The CHI and CHI-FITC were used as markers to confirm the formation of AgCl NPs using their fluorescence properties as well as stabilizers. The fluorescence properties of CHI and CHI-FITC were monitored by a luminescence spectrophotometer, and the morphology of the AgCl NPs was further confirmed by transmission electron microscopy (TEM) and X-ray diffraction (XRD). The fluorescence of CHI and CHI-FITC was quenched by the formation of AgCl NPs, and the Stern–Volmer equation was used to compare the two types of stabilizer. The CHI and CHI-FITC stabilizer were linear and nonlinear, respectively, with respect to the Stern–Volmer equation, and considered to be usable as fluorescence indicators to confirm the formation behavior of AgCl NPs through fluorescence quenching.

## 1. Introduction

Chitosan is a β-1,4-linked polysaccharide of glucosamine (2-amino-2-deoxy-b-d-glucose) with small amounts of *N*-acetylglucosamine, and a natural non-toxic biopolymer derived by the deacetylation of chitin [1,2,3]. Chitosan and chitosan derivatives are attracting attention in the field of biomaterials because of their non-toxicity and biocompatibility [4]. They have abundant amino and hydroxyl groups, and have been studied for drug and gene delivery, hydration gel, tissue engineering, and imaging agents [5,6,7]. Chitosan is not soluble in water or alcohol because it does not break most of the hydrogen bonds between molecular chains, although some amino groups have positive charges in aqueous solution and swell due to the formation of membrane potential. However, chitosan is soluble in an aqueous acidic solution of organic acids such as formic acid, lactic acid, ascorbic acid, acetic acid, or of inorganic acids such as hydrochloric acid. On the other hand, chitosan oligomer (CHI, chito-oligosaccharide) is water-soluble and can be easily prepared by acidic or enzymatic partial hydrolysis of chitosan. Recently, CHI has been studied as a promising material for biomedical applications due to its excellent biocompatibility, biodegradability, antibacterial activity and wound healing effects [6,7,8,9,10].

Nanotechnology is now commonly used in many areas such as cosmetics, electronics, biosensors, pharmaceuticals, and computer science. Nanoparticles (NPs) have been extensively studied for their unique electrical, biological and optical properties, which differ from those of bulk materials [11]. Recently, with the development of nanotechnology, various inorganic NPs such as metals, metal oxides, metal sulfides, and metal chlorides have been successfully synthesized in various ways [10]. Of these, silver chloride (AgCl) is perhaps the most widely recognized and widely used in photographic materials [12], catalyst materials [13], ionic semiconductors [14] and antimicrobial agents [10]. Many methods have been investigated for the synthesis of AgCl NPs using chemical [15,16], radiation [17], and complex methods [18]. However, most of these approaches are environmentally toxic or biologically dangerous, so they are mostly limited in the medical and pharmaceutical fields. Therefore, an environmentally friendly approach to the synthesis of AgCl NPs is essential for its application in a variety of applications.

Therefore, in this study, AgCl NPs were prepared using CHI and CHI derivatives as eco-friendly stabilizers. The formation behavior of NPs was observed using the fluorescence properties of CHI and CHI derivatives reported in previous studies [19].

## 2. Results and Discussion

**Figure 1:**
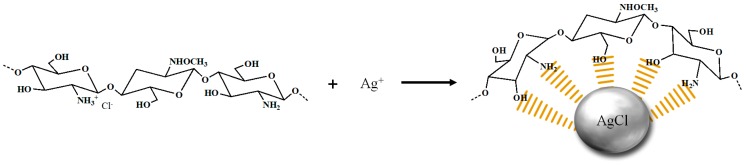
A scheme on the formation mechanism of chitosan oligomer (CHI)/AgCl Nanoparticles (NPs).

**Figure 2:**
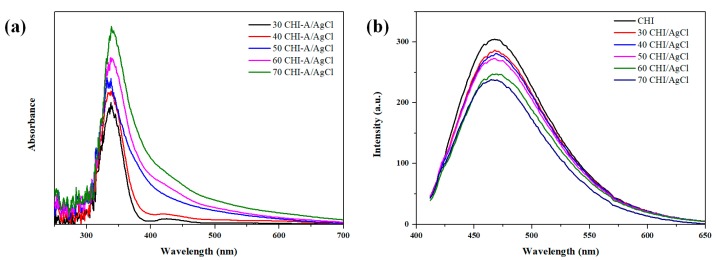

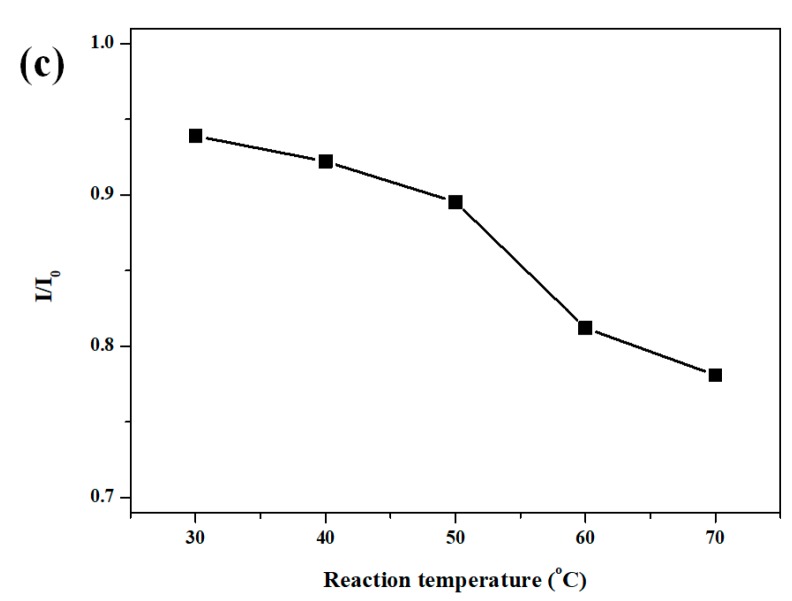
Results of the analysis of CHI/AgCl NPs depending on the reaction temperature; (**a**) UV-Vis spectra; (**b**) photoluminescence (PL) spectra; and (**c**) PL intensities at 475 nm.

The supplied chitosan was decomposed at low molecular weight using enzymes and acids (HCl). The amount of chloride ion (Cl^−^) in chitosan was confirmed by ion chromatography (IC) and, as a result, was 3.2%. The chlorine ions located in the CHI were readily reacted with the Ag ions to form AgCl NPs [20]. Thus, the CHI was used as a source of Cl ions for reducing and stabilization in the NPs formation, and the reduction mechanism of CHI/Ag NPs is illustrated in Figure 1. Thus, chitosan oligomers were used as a source and stabilizer of Cl ions in the nanoparticle formation reaction. The formation of AgCl NPs was confirmed easily by both the color change from bright yellow to yellowish brown and the strong surface plasmon resonance (SPR) peak around 350 nm by UV-Vis spectroscopy. In general, AgCl precipitation occurs, but the prepared NPs solution was almost clear without precipitation because the small and uniform NPs synthesis by the stabilizing agent [10]. A peak corresponding to the surface plasmon resonance (SPR) of AgCl NPs was observed at 350 nm, and the intensity and full width at half maximum (FWHM) increased with increasing reaction temperature (Figure 2a). This increase in FWHM implies an increase in particle size and quantity [10,21]. Figure 2b shows the formation of AgCl NPs according to the reaction temperature through the photoluminescence (PL) of CHI. The PL intensity decreased as the reaction temperature increased. Substituting these results into the Stern–Volmer equation showed a linear behavior corresponding to the static behavior (Figure 2c). As particles are formed, a fluorophore (CHI) reacted with the quencher (Ag) to form an NP complex, which causes a reduction of fluorescence [22,23,24].

**Figure 3:**
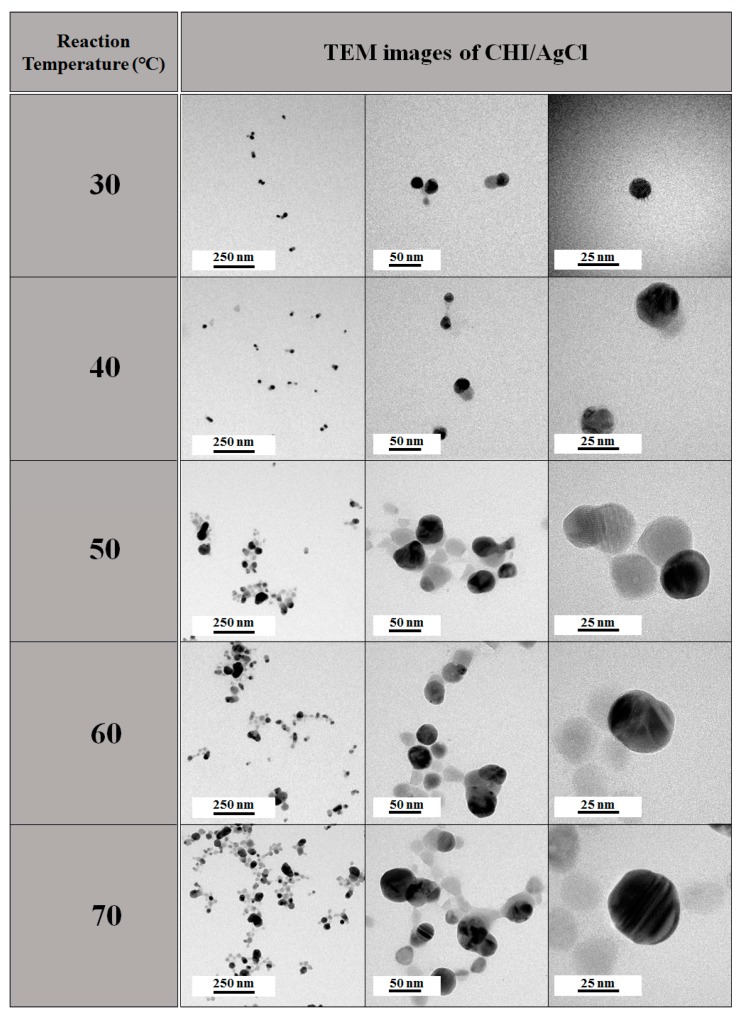
Transmission electron microscope (TEM) images of CHI/AgCl NPs with reaction temperature.

The morphology of the AgCl NPs according to the reaction temperature was analyzed from transmission electron microscope (TEM) images shown in Figure 3. A previous study reported that Ag ions were preferentially reduced by residual Cl ions in CHI to form AgCl nuclei, together with stabilization by amino and hydroxyl groups of CHI [10]. The average diameter (AD) of the NPs increased from 19 to 40 nm with increasing reaction temperature. As the reaction temperature increased, the collision frequency between Ag ions and Cl ions increased. Therefore, the rate of movement and growth of Ag ions to the surface of NPs increased, thereby increasing the particle size of the NPs [10].

**Figure 4:**
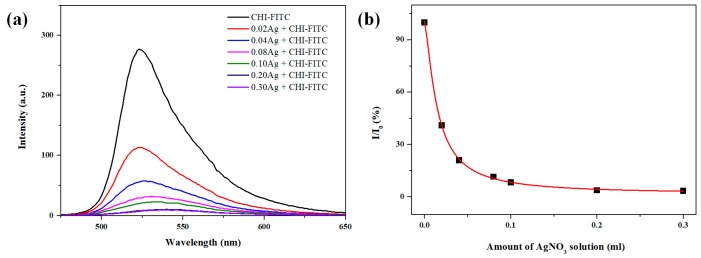
Results of the analysis of chitosan oligomer derivatives (CHI-FITC)/AgCl NPs depending on the concentration of silver nitrate solution: (**a**) PL spectra and (**b**) PL intensities at 520 nm.

The formation behavior of AgCl NPs according to the AgNO_3_ concentration was analyzed using the fluorescence properties of CHI-Fluorescein isothiocyanate (FITC) (Figure 4). It was found that the PL intensity of the CHI/AgCl NPs decreased with an increase in the amount of AgNO_3_ solution. This quenching phenomenon showed a nonlinear behavior when assigned to the Stern–Volmer equation, which is a combination of static quenching and collisional quenching. When compared with the Stern–Volmer plot of the preceding CHI, the slope was higher, indicating a more sensitive fluorescence change [22].

**Figure 5:**
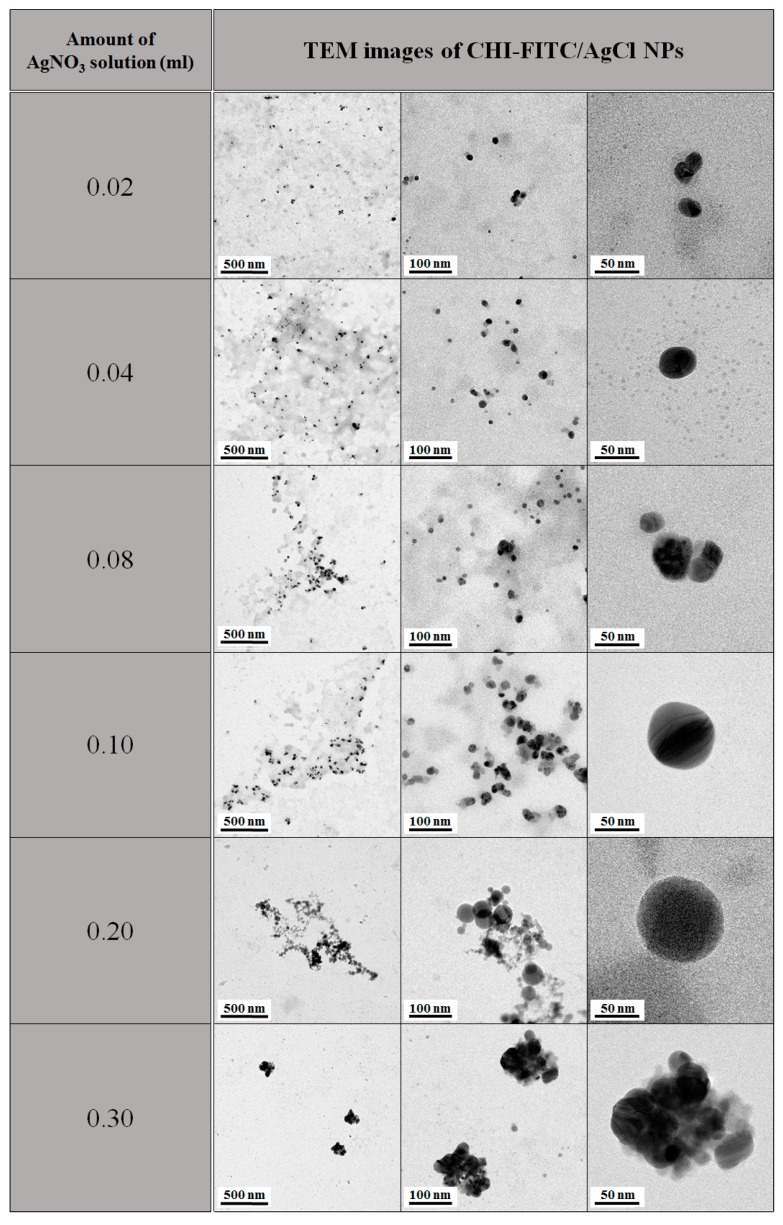
TEM images of CHI-FITC/AgCl NPs with concentration of silver nitrate solution.

Figure 5 shows a TEM image of CHI-FITC/AgCl NPs according to AgNO_3_ concentration. Most of the NPs stabilized with CHI-FITC were spherical, and the particle size increased with increasing AgNO_3_ concentration. However, when the amount added was more than 3.0 mL, coagulation occurred due to the reduction of surface energy caused by the aggregation of particles.

**Figure 6:**
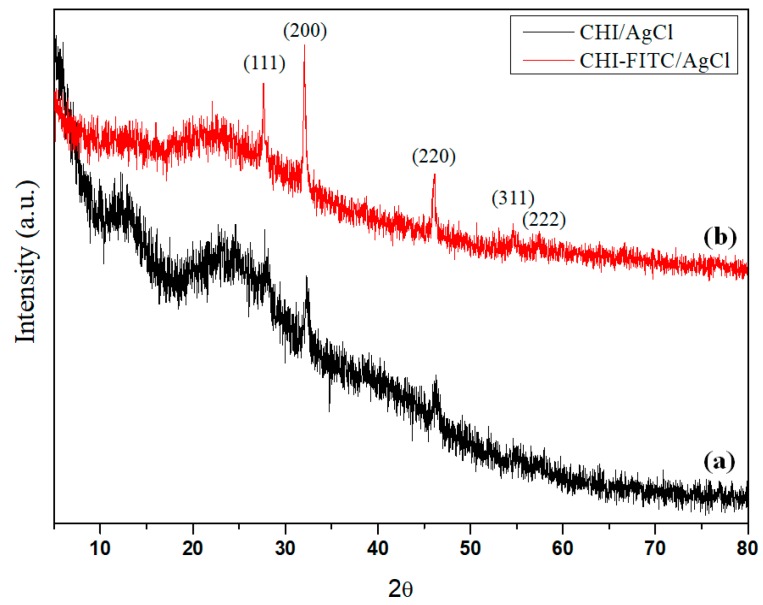
X-ray diffractometer (XRD) Patterns of (a) CHI/AgCl NPs and (b) CHI-FITC/AgCl NPs.

The X-ray diffractometer (XRD) patterns of the CHI/AgCl and CHI-FITC/AgCl NPs are shown in Figure 6. For both samples, a broad peak appeared in the 10–30° range for the amorphous chitosan oligomer. All of the peaks were assigned by the diffraction of crystalline AgCl at 2θ = 27.64, 32.07, 46.06, 54.58 and 57.42°, which correspond to the (1 1 1), (2 0 0), (2 2 0), (3 1 1) and (2 2 2).

**Figure 7:**
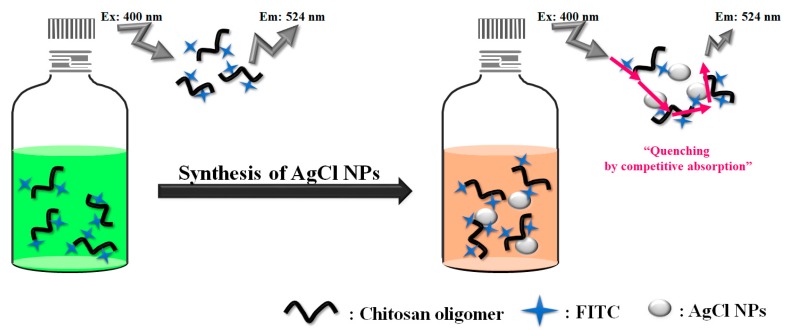
Schematic illustration of the change of PL intensity by preparation of AgCl NPs using CHI-FITC.

Figure 7 shows a schematic diagram of the changes in fluorescence properties of CHI-FITC due to the formation of AgCl NPs. As the AgCl NPs formed, the fluorophore (CHI or CHI-FITC) reacted with the quencher (AgNO_3_) to form an NP complex, resulting in the competitive absorption of FITC with the CHI/AgCl NPs and thus decrease in fluorescence [22,24]. The quenching due to the competitive absorption was confirmed by the absorbance and emission spectra of FITC and CHI/AgCl NPs (data not shown). The degree of PL reduction with the formation of AgCl NPs was higher with CHI-FITC than with CHI. These results demonstrate that the interpretation of the AgCl NPs formation behavior using the fluorescence properties of CHI-FITC is more efficient than using those of CHI.

## 3. Materials and Methods

### 3.1. Materials

CHI was provided by Kittolife Co., (Pyeongteak, Korea). Its degree of deacetylation (DD), amount of Cl ion, and molecular weights were 97%, 3.2%, and ~1000 Da, respectively. Fluorescein isothiocyanate (FITC) was purchased from Sigma-Aldrich Co., (St. Louis, MO, USA). Ethanol (EtOH) and silver nitrate (AgNO_3_) were obtained from Samchun Chemical Co. (purity 99.5%, Pyeongteak, Korea) and Kojima Chemical Co. (purity 99.9%, Sayama, Japan), respectively.

### 3.2. Preparation of CHI/AgCl NPs and CHI-FITC/AgCl NPs Complexes

AgCl NPs stabilized with CHI (CHI/AgCl NPs) were prepared by mixing a CHI solution (2 *w*/*v* %, 30 mL) and an aqueous AgNO_3_ solution (1.7 *w*/*v* %, 0.1 mL). The mixed solution was purged with nitrogen for 5 min to remove oxygen in the solution before starting the reaction. The reaction was then carried out at various reaction temperatures (30, 40, 50, 60, and 70 °C) while stirring. CHI-FITC was used for the preparation of CHI-FITC/AgCl NPs in the previous study [19]. For the synthesis of CHI-FITC, an aqueous solution of CHI was added to an ethanol solution of FITC and the mixture was stirred at room temperature for 24 h in a dark room. After washing with ethanol, centrifugation and vacuum drying were performed to obtain CHI-FITC powder. CHI-FITC/AgCl NPs was prepared by mixing AgNO_3_ solution (1.7 *w*/*v* %) with CHI-FITC solution (0.03 *w*/*v* %, 30 mL) for 8 h after N_2_ purging. At this time, various amounts (0.02, 0.04, 0.08, 0.1, 0.2 and 0.3 mL) of AgNO_3_ were added to examine the effect of AgNO_3_ concentration on the formation of NPs.

### 3.3. Characterizations

The amount of chlorine ion generated by the decomposition of chitosan was analyzed using ion chromatography (IC). Absorption spectra of CHI/AgCl NPs were obtained on a UV-Vis spectrophotometer (UV-2450, Shimadzu, Kyoto, Japan). The formation of the CHI/AgCl NPs and CHI-FITC/AgCl NPs prepared under various conditions were confirmed using a transmission electron microscope (TEM) at 300 kV. X-ray diffractometer patterns (XRD, D8 DISCOVER Bruker AXS, Billerica, MA, USA) were obtained at room temperature with 2θ = 10–80° and a scan rate of 0.5 time/step. The photoluminescence spectra of the CHI/AgCl NPs and CHI-FITC/AgCl NPs were captured using a luminescence spectrophotometer (Varian Cary Eclipse, Varian, CA, USA) equipped with a xenon flash lamp excitation source. Absorption spectra were obtained from the emission spectra of CHI and CHI-FITC at 475 nm and 520 nm, respectively, using an excitation wavelength at 395 nm. In addition, the changes in fluorescent spectra of the CHI/AgCl NPs and CHI-FITC/AgCl NPs complexes according to the reaction temperature and AgNO_3_ concentration were measured using the same luminescence spectrophotometer. In addition, the type of fluorescence quenching was determined using the Stern–Volmer equation [22,25]:

$$\frac{I_{0}}{I}=1+K\left[Q\right]=1+\;k_{q}\tau_{0}\left[Q\right].$$

In this equation, *I*_0_ and *I* are the fluorescence intensities in the absence and presence of a quencher, respectively; *K* is the Stern–Volmer quenching constant; *k_q_* is the bimolecular quenching constant; *τ*_0_ is the unquenched lifetime; and [*Q*] is the quencher concentration.

## 4. Conclusions

In this paper, the formation behavior of AgCl NPs was investigated using the fluorescence properties of CHI and CHI-FITC. The CHI and CHI-FITC served as stabilizers and as indicators of the formation of NPs. For the CHI/AgCl NPs, a linear static quenching behavior was observed when the decrease in fluorescence intensity according to the formation of AgCl NPs was substituted into the Stern–Volmer equation. On the contrary, nonlinear quenching was observed for CHI-FITC/AgCl NPs. In addition, the quenching occurred more rapidly in the CHI-FITC stabilizer than in the CHI stabilizer because the formation of AgCl NPs was structurally affected by the fluorescence properties of FITC. The AgCl NPs obtained by environmentally friendly CHI and CHI-FITC as stabilizers have a considerable potential for use in fluorescence sensors and bio-imaging probes.

## References

[1] Barikani M., Oliaei E., Seddiqi H., Honarkar H. (2014). Preparation and application of chitin and its derivatives: A review. Iran. Polym. J..

[2] Honarkar H., Barikani M. (2009). Applications of biopolymers I: Chitosan. Monatshefte Chem..

[3] Rinaudo M. (2006). Chitin and chitosan: Properties and applications. Prog. Polym. Sci..

[4] Ahmed S., Ikram S. (2016). Chitosan based scaffolds and their application in wound healing. Achiev. Life Sci..

[5] Agrawal P., Strijkers G.J., Nicolay K. (2010). Chitosan-based systems for molecular imaging. Adv. Drug Deliv. Rev..

[6] Kumar M.N.R. (2000). A review of chitin and chitosan applications. React. Funct. Polym..

[7] Pan X., Ren W., Gu L., Wang G., Liu Y. (2014). Photoluminescence from chitosan for bio-imaging. Aust. J. Chem..

[8] Li P., Poon Y.F., Li W., Zhu H., Yeap S.H., Cao Y., Qi X., Zhou C., Lamrani M., Beuerman R.W. (2010). A polycationic antimicrobial and biocompatible hydrogel with microbe membrane suctioning ability. Nat. Mater..

[9] Rabea E.I., Badawy E., Stevens C.V., Smagghe G., Steurbaut W. (2003). Chitosan as antimicrobial agent: Applications and mode of action. Biomacromolecules.

[10] Kang Y.O., Lee T.S., Park W.H. (2014). Green synthesis and antimicrobial activity of silver chloride nanoparticles stabilized with chitosan oligomer. J. Mater. Sci.-Mater. Med..

[11] Zhang X., Liu Z., Shen W., Gurunathan S. (2016). Silver nanoparticles: Synthesis, characterization, properties, applications, and therapeutic approaches. Int. J. Mol. Sci..

[12] Husein M.M., Rodil E., Vera J.H. (2005). A novel method for the preparation of silver chloride nanoparticles starting from their solid powder using microemulsions. J. Colloid Interface Sci..

[13] Wang X., Li S., Yu H., Yu J. (2011). In situ anion-exchange synthesis and photocatalytic activity of Ag_8_W_4_O_16_/AgCl-nanoparticle core–shell nanorods. J. Mol. Catal. A-Chem..

[14] Zhou Z., Long M., Cai W. (2012). Synthesis and photocatalytic performance of the efficient visible light photocatalyst Ag–AgCl/BiVO_4_. J. Mol. Catal. A-Chem..

[15] Dong L., Liang D., Gong R. (2012). In situ photoactivated AgCl/Ag nanocomposites with enhanced visible light photocatalytic and antibacterial activity. Eur. J. Inorg. Chem..

[16] Li L., Zhu Y. (2006). High chemical reactivity of silver nanoparticles toward hydrochloric acid. J. Colloid Interface Sci..

[17] Choi M., Shin K., Jang J. (2010). Plasmonic photocatalytic system using silver chloride/silver nanostructures under visible light. J. Colloid Interface Sci..

[18] Tuncer M., Seker E. (2011). Single step sol-gel made silver chloride on titania xerogels to inhibit *E. coli* bacteria growth: Effect of preparation and chloride ion on bactericidal activity. J. Sol-Gel Sci. Technol..

[19] Lee H.M., Kim M.H., Yoon Y.I., Park W.H. (2017). Fluorescent property of chitosan oligomer and its application as a metal ion sensor. Mar. Drugs.

[20] Gopinath V., Priyadarshini S., Priyadharsshini N.M., Pandianb K., Velusamy P. (2013). Biogenic synthesis of antibacterial silver chloride nanoparticles using leaf extracts of Cissus quadrangularis Linn. Mater. Lett..

[21] Cheon J.Y., Kang Y.O., Park W.H. (2015). Formation of Ag nanoparticles in PVA solution and catalytic activity of their electrospun PVA nanofibers. Fibers Polym..

[22] Lakowicz J.R. (2007). Principles of Fluorescence Spectroscopy.

[23] Kim T.H., Choi M.S., Kwak C.K., Lee J.H., Lee T.S. (2007). Fluorescent Conjugated Polymers as Integrated Sensor Materials. Polym. Sci. Technol..

[24] Green N.J., Pimblott S.M., Tachiya M. (1993). Generalizations of the Stern–Volmer relation. J. Phys. Chem..

[25] Htun T. (2004). A negative deviation from Stern–Volmer equation in fluorescence quenching. J. Fluoresc..

